# Structuring medication signeturs as a language regression task: comparison of zero- and few-shot GPT with fine-tuned models

**DOI:** 10.1093/jamiaopen/ooae051

**Published:** 2024-06-18

**Authors:** Augusto Garcia-Agundez, Julia L Kay, Jing Li, Milena Gianfrancesco, Baljeet Rai, Angela Hu, Gabriela Schmajuk, Jinoos Yazdany

**Affiliations:** Division of Rheumatology, University of California San Francisco, San Francisco, CA 94110, United States; Division of Rheumatology, University of California San Francisco, San Francisco, CA 94110, United States; Division of Rheumatology, University of California San Francisco, San Francisco, CA 94110, United States; Division of Rheumatology, University of California San Francisco, San Francisco, CA 94110, United States; Division of Rheumatology, University of California San Francisco, San Francisco, CA 94110, United States; Division of Rheumatology, University of California San Francisco, San Francisco, CA 94110, United States; Division of Rheumatology, University of California San Francisco, San Francisco, CA 94110, United States; Division of Rheumatology, University of California San Francisco, San Francisco, CA 94110, United States

**Keywords:** natural language processing, large language models, in-context learning, language regression, immunomodulating drugs

## Abstract

**Importance:**

Electronic health record textual sources such as medication signeturs (sigs) contain valuable information that is not always available in structured form. Commonly processed through manual annotation, this repetitive and time-consuming task could be fully automated using large language models (LLMs). While most sigs include simple instructions, some include complex patterns.

**Objectives:**

We aimed to compare the performance of GPT-3.5 and GPT-4 with smaller fine-tuned models (ClinicalBERT, BlueBERT) in extracting the average daily dose of 2 immunomodulating medications with frequent complex sigs: hydroxychloroquine, and prednisone.

**Methods:**

Using manually annotated sigs as the gold standard, we compared the performance of these models in 702 hydroxychloroquine and 22 104 prednisone prescriptions.

**Results:**

GPT-4 vastly outperformed all other models for this task at any level of in-context learning. With 100 in-context examples, the model correctly annotates 94% of hydroxychloroquine and 95% of prednisone sigs to within 1 significant digit. Error analysis conducted by 2 additional manual annotators on annotator-model disagreements suggests that the vast majority of disagreements are model errors. Many model errors relate to ambiguous sigs on which there was also frequent annotator disagreement.

**Discussion:**

Paired with minimal manual annotation, GPT-4 achieved excellent performance for language regression of complex medication sigs and vastly outperforms GPT-3.5, ClinicalBERT, and BlueBERT. However, the number of in-context examples needed to reach maximum performance was similar to GPT-3.5.

**Conclusion:**

LLMs show great potential to rapidly extract structured data from sigs in no-code fashion for clinical and research applications.

## Introduction

Prescription signeturs (sigs), Latin for “let it be labeled,” refer to the text describing medication instructions as written by the prescribing physician and printed on the medication container. They contain specific directions as to the time, frequency and number of pills the patient should take (eg, “take one tab after every meal”). Most sigs are straightforward and specific (eg, “take one 40mg tab per day”), but some sigs can contain complex instructions due to tapering regimens or narrow toxicity profiles (eg, “take one tab m-f, two tabs on weekends”; “take 4 tabs/day for a week, then 3 tabs/day for another week, then 2 tabs/day”).

Additionally, sigs often express the same information in different ways (eg, “take 200mg every day” and “take one tablet by mouth every day”) or have some ambiguity: from having general indications that are difficult to turn into a single structured variable (eg, “take 1-3 pills a day”), to having contradicting instructions due to presenting a generic sig that the physician has replaced without overwriting the original description (eg, “take 1 pill a day. Take 2 pills a day”). Furthermore, some are unresolvable without additional chart review (eg, “as directed”).

Implementing efficient methods to accurately extract structured data from sigs is essential to, in turn, have reliable structured data from which to conduct downstream research and make clinical decisions, such as adjusting dosage for a current patient based on toxicity or conducting large-scale studies to establish safety in real world settings. While manual annotation is still the gold standard, different approaches have been studied to address this challenge. In this work, we focused on methods to extract the average daily dose for a medication. We believe this task is particularly challenging because it requires both named entity recognition (NER) as well as algebraic operations with the identified entities (eg, calculating the average dose per day of the week and then computing a total average), which we refer to as language regression throughout this manuscript.

Methods for this task can be primarily divided into 2 groups. The first group consists of designing a hardcoded or rule-based tool. This is usually based on reducing sigs to regular expressions from which a formula can be deducted. While highly accurate for the dataset they are created for, these will fail to generalize to unseen datasets and are significantly time-intensive, as they require manual review, textual pre-processing and regular expression design. Examples include the R package *Doseminer*^1^ or sig2db^2^. The second group consists of using some form of classifier. This approach consists of using NER or contextual embeddings either fully fine-tuned or paired with a fine-tuned classifier head. While much less time-intensive than hardcoded tools, these models require training with a large variety of sigs and thus do not remove the need for manual annotation. They will also fail to generalize to unseen output values.^3^^,^^4^

A more adaptable approach is to use a large language model (LLM), ideally somewhat proficient in question answering, information extraction, and basic mathematical operations, such as GPT.^5^ In this way, LLMs can be queried for specific structured data fields that derive from sigs. This approach has two advantages: (1) it maximizes model versatility, as it will be the easiest approach to generalize, especially if research finds minimal or no fine-tuning is needed, and (2) it will minimize, or ideally eliminate, the need for manual annotation. This approach was already described and prototypically implemented, albeit with older LLM architecture, in previous research.^6^ However, recent LLMs such as GPT have shown drastic advances for medical reasoning^7^ and information extraction,^8–10^ offering a no-code solution to this problem. The viability of using LLMs to extract structured data explicitly available in free-text clinical notes (information extraction) has been widely discussed,^11^ as have its limitations.^12^ While this feat can greatly facilitate applications requiring these data, such as clinical decision support or research, its viability for language regression is still unclear,^13^ as is the varying contribution of in-context examples.^14^

To assess the feasibility of applying LLMs to analyze sigs, their overall performance needs to be established. Additionally, a comparison with a smaller, fine-tuned model, should be performed. Our study aims to do so by providing the following innovations: (1) examining the performance of LLMs to structure complex sigs, which can be framed as a language regression task that requires simple mathematical operations such as products, sums, and averages, (2) evaluating the minimum number of manual annotations, provided as in-context examples, to achieve maximum performance, and (3) providing a comparison with a baseline consisting of smaller, fine-tuned models, such as ClinicalBERT^15^ or BlueBERT.^16^ In this study, sigs from two commonly prescribed immunomodulating drugs that commonly have complex instructions were used to conduct these analyses.

## Methods

### Dataset

The data used in this study were derived from data aggregated from 2 other observational studies examining medication dose. Briefly, one study was based at an academic medical center (UCSF Health) and involved all hydroxychloroquine orders issued from the rheumatology or dermatology clinics for adult patients, between 2015 and 2020. This yielded 702 hydroxychloroquine 200 mg sigs included in the current study, representing approximately 12 250 orders from 3000 patients.

Separately, a second study used data from Rheumatology Informatics System for Effectiveness, a national electronic health record (EHR)-based registry with data derived from over 300 practices^17^ and involved all oral glucocorticoid orders for patients with rheumatoid arthritis and Medicare insurance during 2018. This yielded 22 104 sigs for prednisone or its equivalents included in the current study, representing approximately 194 500 orders from 44 500 patients.

Our initial annotation (referred to as original annotation) was performed by three separate annotators for hydroxychloroquine (one epidemiologist specializing in rheumatology and two rheumatology senior research data analysts) and five for prednisone (two rheumatologist attending physicians, one rheumatology fellow, one internal medicine resident, and one senior research data analyst). In case of disagreement, the senior rheumatologist’s opinion was included. For the hydroxychloroquine set, if two conflicting instructions were found, chart review of clinical notes was performed, which confirmed that the second instruction in a series was correct. These datasets contain complex expressions, such as:

- EHR default text overwriting (eg, “take 1 tablet by mouth daily. take 1 and 1/2 tablets by mouth daily”). For this type of sig all manual annotators only considered the second instruction, based on the results of the chart review mentioned above.
- Ranges (eg, “1.0 mg tabs. take 1-5 milligram by oral route every day”)
- Complex patterns (eg, “take 1 tablet every tu, wed, th, sat., sun. take 2 tablets mon and fri only”)

### Overall approach

Three models were tested in the hydroxychloroquine set, including (1) a ClinicalBERT^15^ and BlueBERT^16^ sequence classification model fine-tuned for regression on 75% of the dataset and tested on the remaining 25%, (2) zero-shot GPT-3.5 and GPT-4, and (3) few-shot (1,5,10,20,40,60,75,100) GPT-3.5 and GPT-4. For dataset splitting, stratification was used to make sure both splits have the same output label distribution as the full set.

### In-context examples for few-shot learning

For the few-shot approach, in-context examples were provided with four different criteria. We conceived these criteria as potential methods to sample highly informative sigs for in-context learning for minimal manual annotation, as follows:

- Selected randomly from the full set of sigs (labeled *random*);
- Selected randomly from the top 15% by sig length (labeled *word count*);
- Selected randomly from the top 15% by infrequent words (labeled *uncommon words*); and
- Selected randomly from the top 15% by absolute distance between zero-shot GPT-3.5 and GPT-4’s prediction (labeled *model disagreement*).

In all selective approaches, we sampled from the top 15%, as opposed to selecting the longest or most complex sigs in the set. By opting to sample from the top 15%, the aim was to create a subset that may better represent potentially unseen sets that could exhibit slight deviations from our distribution. For tests on the full set, sigs that are provided as in-context examples are removed from the list of prompts. After obtaining results for the hydroxychloroquine dataset, the best-performing model from all zero- and few-shot GPT-based approaches was compared with a similarly fine-tuned ClinicalBERT model for the larger prednisone set.

### Model selection and construction

For all GPT models, a temperature of 0.5 and a max output token length of 10 with the API version 2023-05-15 were used. The most recent version of both models was used at the time of the experiment (November 13-15, 2023). As a comparison baseline, we selected ClinicalBERT and BlueBERT in its PubMed + MIMIC-III version. Both of these models are BERT based and have been pretrained with medical papers and free-text notes and should perform reasonably given a sufficiently large dataset and fine-tuning.

For ClinicalBERT, we used a learning rate of 1E−5, ADAM optimization and mean squared error loss. When designing this study, we considered offering the output of a locally hosted chat model, Llama2-13B or Llama2-7B, as an additional result. However, significant model verbosity was observed, as the output would be contaminated by the model making up additional examples or adding undesired explanations that would make the task of extracting the model’s numeric output from its textual reply another natural language processing task in itself. This led us to discard this option. For prednisone sigs, as tab sizes vary, the tab size was included in the sig (eg, “4.0 mg tabs. take 1-3 tablets by mouth daily”). The model prompt always followed the same structure:

“The following text describes the dosing pattern of [Medication name]. The task is to extract the daily average dose in number of milligrams per day. Reply only with a number with one decimal value. Do not include an explanation. Examples: [Input 1]. [Output 1]. (…) [Input n]. [Output n]. [Input].”

**Figure 1.**
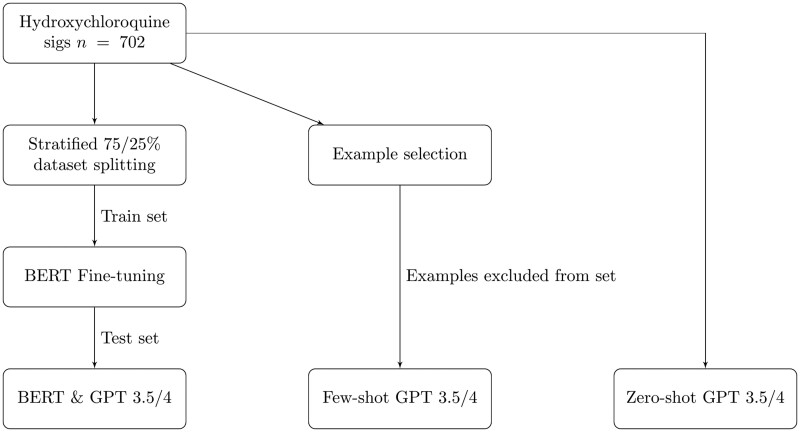
Experiment flowchart for hydroxychloroquine.

**Figure 2.**
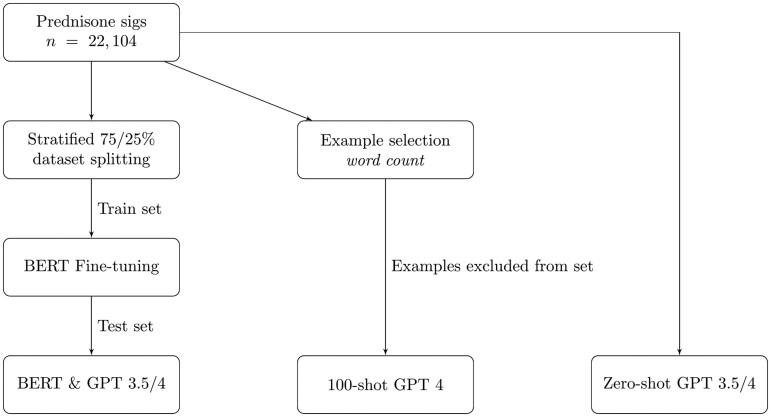
Experiment flowchart for prednisone.

A flowchart describing the experiments for both datasets is presented in Figures 1 and 2.

### Outcomes

The outcome of interest was the proportion of sigs within a certain threshold of absolute error in miligrams per day. A range of errors, based on clinical significance, was chosen between a maximum of 10% (highly clinically significant) and 0.1% (not clinically significant) of the average daily dose for each drug. Mean absolute error (MAE) and standard deviation (SD) were calculated as well. In each case, we reported results for the complete set (for zero- and few-shot settings) as well as for the selected 25% test set to offer a fair comparison between the fine-tuned model and other approaches.

### Error analysis

Following these experiments, we conducted an error analysis procedure on the subset of sigs for which the best-performing model’s annotation disagreed with the original annotation. For those sigs, we requested two additional annotators (B.R. and A.H., both rheumatologists, anonymized as annotator 1 and annotator 2) to re-annotate said sigs. Inclusive of providing an annotation in mg/day, when possible, annotators were asked to provide two additional and not mutually exclusive outputs:

- An indication that the sig was ambiguous (ie, two contradicting instructions or a range) and
- An indication that the sig was impossible to resolve (ie, not enough information to provide an estimate, or the sig contains incorrect information, eg, a mention of ml or a different medication such as methotrexate, or “as directed”).

For this error analysis task, we asked both annotators to re-annotate the hydroxychloroquine set to estimate interrater agreement, and we divided the prednisone set evenly. Instructions given to these two annotators were identical to those of the original annotation, including an indication to resolve two conflicting instructions by attending to only the second one. We reduced the size of the prednisone set for manual annotation by removing all but one entry where the manual annotation and the model annotation were identical, and thus the difference between both was the same. Our selected outcomes for error analysis, which are thus also not mutually exclusive, are as follows:

- Number of sigs marked as ambiguous;
- Number of sigs marked as impossible to resolve;
- Annotator 1 or 2 disagrees with original annotation; and
- Annotator 1 or 2 disagrees with model’s annotation and agrees with original annotation. We define these as confirmed model errors.

We provide Cohen’s kappa as a measure of interrater agreement for the error analysis outcomes in the hydroxychloroquine set, and the MAE and SD comparing annotators 1 and 2’s dose values with the original annotation and the model.

### Ethics

Sigs were de-identified and no PHI was included. The GPT-4 and GPT-3.5 models were used via the HIPAA compliant Versa API at UCSF Health such that no data were either permanently transferred to or stored by either Microsoft or OpenAI for any purposes. BERT experiments were conducted on a local computer. The data derive from source studies with IRB numbers 16-21347 for hydroxychloroquine and 21-34133 for prednisone.

## Results

**Figure 3.**
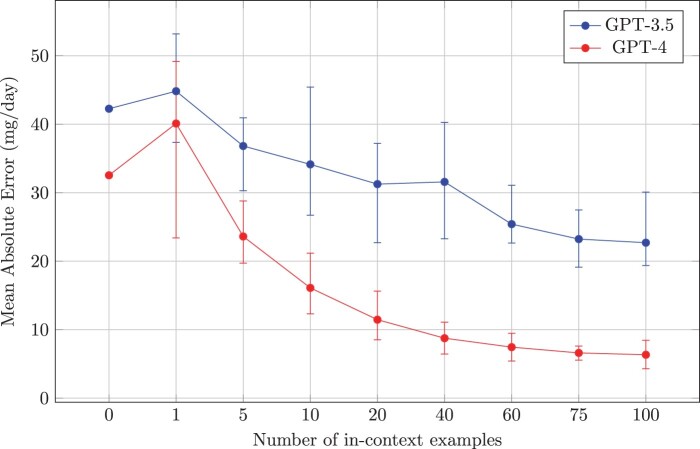
Mean absolute error of hydroxychloroquine sigs. Error bars correspond to maximum and minimum values for number of in-context of examples across criteria. Results for the full set are only possible with GPT models, a comparison with BERT models on the test set is provided in Table 1.

**Figure 4.**
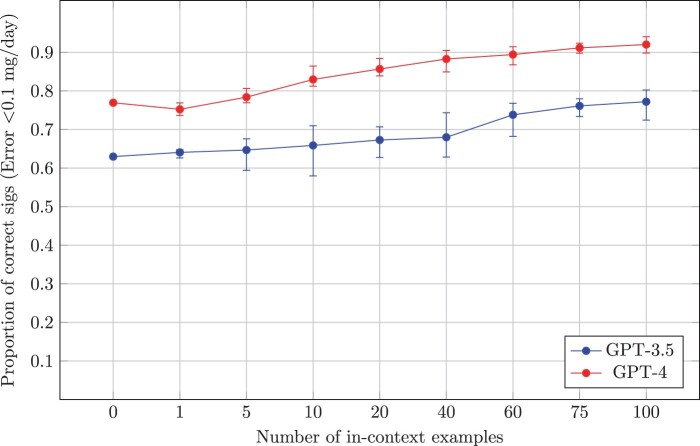
Proportion of correctly annotated hydroxychloroquine sigs. We define correct as MAE <0.1 mg/day. Error bars correspond to maximum and minimum values for number of in-context of examples across criteria. Results for the full set are only possible with GPT models, a comparison with BERT models on the test set is provided in Table 1. MAE = mean absolute error.

Results on the hydroxychloroquine set for GPT-3.5 and GPT-4 for the full set are presented in Figures 3 and 4, while results comparing GPT models with BERT models on the 25% designated test sets are provided in Table 1. All GPT models vastly outperformed BERT models, even in a zero-shot setting. Both GPT-3.5 and GPT-4 experienced a proportional accuracy gain as more context is provided, with GPT-4 consistently annotating 15% more sigs within an error threshold of 0.1 mg/day or less. GPT-4 plateaud at 94.0% sigs within 0.1 mg/day or less and an MAE (SD) of 4.3(±22.2) mg/day at 100 in-context examples, from its zero-shot performance of 76.9% and 32.5(±146.1) mg/day. Complete experiment results are provided in Appendix A. A slight advantage was observed in all in-context example selection criteria over random selection.

**Table 1.** Performance of fine-tuned BERT models, compared to different GPT versions, on the hydroxychloroquine and prednisone test sets.

Model	MAE	SD	Within 0.1%	0.5%	1%	5%	10%
**Hydroxychloroquine**	mg/day	mg/day	% sigs	% sigs	% sigs	% sigs	% sigs
ClinicalBERT	78.87	83.28	0.0%	0.0%	28.2%	35.6%	36.2%
BlueBERT	29.58	55.5	2.8%	14.1%	21.5%	75.1%	77.4%
GPT-3.5 Zero-shot	45.02	89.37	63.8%	63.8%	63.8%	65.0%	66.9%
GPT-4 Zero-shot	19.50	59.33	75.6%	76.9%	77.5%	80.0%	82.5%
GPT-3.5 100-shot	24.79	53.38	76.1%	76.1%	76.1%	76.8%	78.2%
**GPT-4 100-shot**	**5.77**	**28.67**	**94.4%**	**94.4%**	**94.4%**	**94.4%**	**95.0%**
**Prednisone**							
ClinicalBERT	1.04	23.56	0.5%	3.7%	8.4%	68.4%	96.3%
BlueBERT	0.99	23.68	1.5%	9.4%	16.2%	79.0%	96.7%
GPT-3.5 Zero-shot	3.49	21.80	68.4%	70.5%	70.8%	71.4%	72.4%
GPT-4 Zero-shot	0.99	23.55	81.8%	87.9%	88.4%	88.9%	89.9%
**GPT-4 100-shot**	**0.75**	**20.57**	**93.0%**	**94.3%**	**94.4%**	**94.6%**	**94.8%**

Hydroxychloroquine *n* = 702, prednisone *n* = 22 104. In-context examples provided through the *word count* criterion, that is, selected randomly from the top 15% by sig length. Within % refers to within % of average dose. Average dose of hydroxychloroquine: 298 mg/day. Average dose of prednisone: 9 mg/day. Due to cost limitations, we were unable to include GPT-3.5 100-shot for prednisone. Bold values indicate the best performing model.

Abbreviation: MAE = mean absolute error.

**Figure 5.**
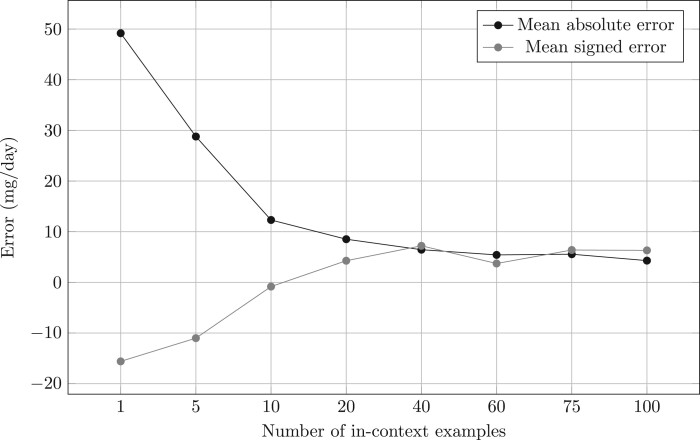
MAE and signed error for GPT-4 word count. While signed error plateaus at 40 in-context examples, absolute error does not. MAE = mean absolute error.

The best-performing approach was GPT-4 with the 100-shot *word count* criterion. Appendix A displays the distribution of this approach’s errors across the distribution of output doses. For this approach, errors were highly concentrated in infrequent output doses that have the most complex sigs associated to them, as discussed in the error analysis section of our experiments. As an additional insight into model errors, we present the evolution of signed and absolute error with different amounts of in-context examples for the *word count* method in Figure 5. While signed error plateaud at 40 in-context examples, absolute error did not.

As described above, based on the results of the experiments shown in Appendix A, we selected the 100-shot *word count* GPT-4 model to compare to manual annotation and fine-tuned BERT-based models on the prednisone sigs. For the complete set, GPT-4 achieved an MAE of 0.53 (±10.63) mg/day and 94.5% sigs within 0.1% of the average daily dose.

We analyzed errors in the predictions for hydroxychloroquine and prednisone, detailed in Tables 2 and 3, respectively. This involved examining instances where our model’s annotation differed from the original annotation, resulting in 36 instances for hydroxychloroquine and 267 for prednisone. For hydroxychloroquine, we reviewed all 36 instances. For prednisone, we only included one instance of each group of instances where the model’s annotation and the original annotation are identical (and in disagreement). Both drugs had sigs with ambiguity per our annotators. Most discrepancies were found to be model errors, as our annotators aligned with the original annotation against the model’s prediction. However, a few instances were identified as mislabeled, where annotators favored the model’s annotation over the original. Further details on the error analysis for hydroxychloroquine can be found in Appendix B.

**Table 2.** Manual annotation of model errors in hydroxychloroquine sigs (*n* = 36).

Hydroxycholoroquine (*n* = 36)	Annotator 1 *n*	Annotator 2 *n*	Cohen’s kappa
Sig is ambiguous^a^	14	13	0.8
Sig cannot be resolved^a^	2	2	0.5
Disagree with original annotation^a^	12	16	0.8
Confirmed model error^a^	24	20	0.8
Original annotation MAE (SD) (mg/day)	25.7 (±55.3)	27.3 (±54.8)	N/A
Model MAE (SD) (mg/day)	46.3 (±54.0)	46.7 (±54.1)	N/A

When both annotated, annotators disagree on 4 annotations with MAE 1.6 mg/day. Confirmed model error is defined as annotator 1/2 agreeing with original annotation and disagreeing with model annotation. Full table, including sig content, is provided in Appendix B.

Abbreviation: MAE = mean absolute error.

a Categories are not mutually exclusive.

**Table 3.** Manual annotation of model errors in predisone sigs (*n* = 267).

Prednisone (*n* = 267)	Annotator 1 *n*	Annotator 2 *n*
Sig is ambiguous^a^	113	191
Sig cannot be resolved^a^	66	227
Disagree with original annotation^a^	16	26
Confirmed model error^a^	164	93
Original annotation MAE (SD) (mg/day)	0.25 (±1.3)	6.40 (±24.8)
Model MAE (SD) (mg/day)	1.83 (±1.4)	28.13 (±144.5)

Model error is defined as annotator 1/2 agreeing with original annotation and disagreeing with model annotation.

Abbreviation: MAE = mean absolute error.

a Categories are not mutually exclusive.

## Discussion

Establishing the accurate prescribed dose of a medication is required for clinical research studies and federal quality reporting programs. Traditionally, arriving at an accurate prescribed dose requires manual annotation for medications with complex sigs. This can be costly and time-consuming. In this paper, the performance of GPT-3.5, GPT-4, ClinicalBERT, and BlueBERT for this task was evaluated. It was found that GPT-4 vastly outperforms all other models. However, depending on error tolerance, BlueBERT may offer a viable solution for a fraction of the cost. In addition, we found that providing annotated sigs as in-context examples significantly increases model accuracy. These findings align with the existing literature supporting the potential of GPT as an excellent method for information extraction, and contribute new sights into its clinical language regression capabilities. However, our findings also highlight an area with potential for improvement, as the model required a significant number of annotated sigs before it reached its maximum performance.

When selecting criteria to provide the most informative samples for in-context learning, it was found that any of the methods we considered (selecting particularly long sigs, or sigs with uncommon words, or sigs where zero-shot GPT-3.5 and GPT-4 disagree the most) slightly outperformed random selection. Further research using larger datasets and additional medications is required to fully understand which of these methods, or any other, works best. However, by designing our criteria selecting among the top 15% of examples as opposed to simply choosing the most suitable ones, we believe our results will generalize to other domains as well, and selection criteria for in-context learning will outperform random selection.

Our findings can be interpreted in the broader context of the performance of LLMs for classification and regression. Using LLMs for classification has shown that a highly relevant factor for successful in-context learning is to provide a distribution that is most representative of the dataset distribution, even if incorrect examples are intentionally provided.^18^ However, if the goal was to select unannotated sigs to maximize model performance, it would be unfair to use the annotated value as a factor in that selection. An additional interesting finding is that, despite the accuracy values of GPT-4 being consistently higher than those of GPT-3.5, the performance gain rate with added context for both models is similar, although MAE values for GPT-3.5 across example criteria are noisier, as depicted in Figure 3.

We anticipated GPT-4 outperforming GPT-3.5, as it is a larger model trained on more data. However, the fact that it needs the same amount of context to converge to its maximum dataset performance is somewhat surprising. This result can be explained by a broader discussion of the limitations of LLMs when doing mathematical operations, as models show limitations for mathematical reasoning.^13^ Even at its maximum performance values, the model struggled with a small number of sigs. Error analysis and reannotation revealed that these sigs are often ambiguous, and some, but not all errors would also be committed by a manual annotator with domain knowledge. An aspect of our results that we consider particularly relevant is the presence and resolution of ambiguous sigs. Mostly, these were generated when a sig is automatically included by default in the EHR, and the prescribing physician, instead of overwriting it, added a second duplicative sig. This results in a particularly common pattern that is present in approximately half of the model’s errors for hydroxychloroquine (eg, “take 1 tablet by mouth daily. take 1 and 1/2 tablets by mouth daily”). The original annotators resolved this ambiguity by ignoring the first set of instructions based on clinical note reviews that revealed the second sig was what the prescriber intended. Almost all models, however, not knowing which of the instructions was the correct one, simply tended to calculate the average of both sets, as is the case when the sig offers a range instead of a set value. The models also often incur errors in ranges, which can in some cases be very wide (eg, “take 1-8 tablet by oral route every day”), making the impact of this error in MAE results significant. We intentionally decided to not add any specific instructions to the model to address these issues to provide results that are consistent with a minimal (or no) manual annotation scenario. However, the metrics presented in this manuscript could be further improved by simply adding specific instructions to the model’s prompt.

Our study has several important limitations. First, resource limitations meant we were unable to run all experiments in our larger prednisone set. While the model provides excellent performance in a 100-shot setting, it may be that a higher number of in-context examples may offer even better results, or that less context performs similarly well. Second, our data were extracted from an academic center and a national rheumatology registry that represents about 30% of the US rheumatology workforce.^17^ Together these data sources include hundreds of rheumatologists practicing in academic centers and community-based clinics; however, the data may not be representative of sigs written by other providers, including those outside of the United States or nonrheumatologists. Third, the limited size of our hydroxychloroquine set may have limited ClinicalBERT’s and BlueBERT’s performance, considering that their error rate is significantly higher than that of prednisone as presented in Table 1. For this reason, if only very few sigs are available, GPT will be a far superior approach. Finally, our study does not evaluate the performance of these models for other drugs in which a larger distribution of potential output values may exist.

In our work, GPT-4 exhibited superior accuracy extracting average daily doses from sigs without the need for extensive coding or prolonged computational time. However, implementing this method at scale may prove financially challenging. For instance, during our experiments, the cost was calculated at $0.03 per 1000 tokens for input and $0.06 per 1000 tokens for output, which, despite seeming minimal, escalates quickly with tasks requiring large contexts or numerous prompts. The total approximate cost of our experiments, including some repetitions, was around $3700. In contrast, BlueBERT emerges as a much more cost-effective alternative, although it may not match GPT-4’s performance in all aspects. In the short term, focusing on improving smaller models like these may offer a financially viable pathway for enhancing clinical decision support and research depending on error tolerance if sufficient data for training are available. Future authors may also consider smaller, locally deployable LLMs based on platforms such as Llama-2 that will likely increasingly show competitive performance in algorithmic reasoning and reduced verbosity, as measured by datasets such as GSM8K.^19^

In sum, we believe that this study highlights the potential of general purpose LLMs to significantly reduce the burden of manual data annotation by efficiently and accurately turning EHR unstructured data into structured data with minimal supervision and annotation. While it seems that LLMs have not fully eliminated the need for manual annotation if very high accuracy is needed, they are a very significant step forward toward minimizing it. Application of these methods will ultimately make the data more useful for research and clinical care by accelerating the research and clinical decision support pipelines. In the future, we aim to augment the sig extraction pipeline by standardizing chart review of clinical notes as a second step to resolve ambiguous or unresolvable sigs and using this information to improve prescriber sigs. We also hope future work will reveal other factors useful for more selective in-context learning to reduce costs.

## Supplementary Material

ooae051_Supplementary_Data

## Data Availability

Scripts for OpenAI models and the BERT experiment pipeline are available at https://github.com/AugustoGarcia/llm_sig_annotation.
