# Supplementary material for: Structuring medication signeturs as a language regression task: comparison of zero- and few-shot GPT with fine-tuned models
Source: JAMIA Open. 2024 Jun 18;7(2):ooae051. doi: 10.1093/jamiaopen/ooae051 (PMC11195626; doi:10.1093/jamiaopen/ooae051)
Supplement: ooae051_Supplementary_Data [file ooae051_supplementary_data.zip › Appendix B.pdf]

Appendix B: Hydroxychloroquine error analysis

Table 6: **hydroxychloroquine error analysis for GPT-4 100-shot *word count***. All values are mg/day. OA = Original Annotation, A1 = Annotator 1, A2 = Annotator 2

Sig	Model	OA	A1	A2
take 2 tablets by mouth twice daily monday-friday, then 1 tablet by mouth on weekend days.	571.4	342.9	342.9	342.9
take 1 tablet(200 mg) by mouth once daily	200	400	200	200
take 2 tablets (400 mg total) by mouth daily. take 1 tablet by mouth daily	400	200	200	200
take 7.6 mls (190 mg total) by mouth 2 (two) times daily.	380	190	380	380
alternate 1 tablet every other day and 1/2 tablet every other day.	150	300	150	150
take 1 tablet (200 mg total) by mouth daily. 1 tab bid m-f, hold on sat/sun	285.7	142.9	285.7	285.7
take 1 tablet (200 mg total) by mouth twice a day. m-f only	400	285.7	285.7	285.7
take 1.5 tablets (300 mg total) by mouth daily. 200 mg daily	300	200	200	200
take 1.5 tablets (300 mg total) by mouth daily. take 2 tablets by mouth daily	500	400	400	400
take 1.5 tablets (300 mg total) by mouth daily. take one tablet by mouth once daily	300	200	200	200
take 1/2 tablet (100mg) by mouth every morning and 1 tablet (200mg) in the evening	214.3	300	300	300
take 5ml once daily by mouth.	200	125		
1 pill twice per day on weekdays, and 1 pill daily on weekends.	285.7	342.9	342.9	342.9
m-f take 200 mg bid and sat/sun 200 mg	285.7	342.9	342.9	342.9
please take 1 pill bid m-f and 1 pill qd sat/sun	285.7	342.9	342.9	342.9
take 1 tablet po bid x 5 days/week (m-f), 1 tablet po qd x 2 days/week (sa/su)	285.7	342.9	342.9	342.9
take 1 pill once daily 5 days a week	200	142.9	142.9	142.9
take 2 pills on sunday and 1 pill all other days	285.7	228.6	228.6	228.6
take 1 tablet by mouth daily	200	250	200	200
take 1-1.5 tablets (200-300 mg total) by mouth daily.	250	300	250	250
take 1.5 tablets (300 mg total) by mouth daily. 200 mg on odd days and 400 mg on even days	350	300	300	314.3
take 1 tablet (200 mg total) by mouth daily. take 2 tables every 4th day.	200	242.9	242.9	257.1
take 1 tablet (200 mg total) by mouth daily. take 2 tablets every 4th day.	200	242.9	242.9	257.1
take 3/4 tablet by mouth every day for your discoid lupus	150	187.5	150	150
400mg daily m/w/f and 200mg daily t/th/sat/sun	314.3	285.7	285.7	285.7
take 2 pills monday, tues, thurs, fri. take 1 pill wed, sun, sat	285.7	314.3	314.3	314.3
take 1 tablet (200 mg total) by mouth daily. 2 pills monday-friday, 1 pill saturday-sunday	314.3	342.9	342.9	342.9
take 1 tablet by mouth twice daily, except 1 day of the week take 1 tablet only.	371.4	342.9	371.4	371.4
take 1 tablet every tu, wed, th, sat., sun. take 2 tables mon and fri only.	285.7	257.1	257.1	257.1
take 2 pills on monday, wednesday and friday and 1 pill on every other day.	257.1	285.7	285.7	285.7
take one tablet once daily monday through thursday. take two tablets once daily friday through sunday.	285.7	257.1	285.7	285.7
take 1 tablet twice a day except take 1 tablet on sunday	342.9	371.4	371.4	371.4
take 200mg bid 6 days per week and 200mg daily one day per week.	342.9	371.4	371.4	371.4
take one tablet by mouth every three days	66.7	57.1	71.4	71.4
take 1/2 tablet every other day	50	57.1	50	50
take one tablet daily, and 2 tablets every 4th day.	250	242.9	242.9	257.1
