# Supplementary material for: Structuring medication signeturs as a language regression task: comparison of zero- and few-shot GPT with fine-tuned models
Source: JAMIA Open. 2024 Jun 18;7(2):ooae051. doi: 10.1093/jamiaopen/ooae051 (PMC11195626; doi:10.1093/jamiaopen/ooae051)
Supplement: ooae051_Supplementary_Data [file ooae051_supplementary_data.zip › Appendix A.pdf]

Appendix A: Results of all hydroxychloroquine experiments

Table 4: **Distribution of errors across sig doses for GPT-4 100-shot *word count*.**

Dose (mg/day)	Sigs (n)	Errors (n)
300	168	4
400	138	0
200	106	3
285.71	35	3
342.86	33	7
100	17	0
85.71	13	0
257.14	12	2
250	10	1
150	10	0
142.86	9	2
600	7	0
800	5	0
371.43	5	2
125	3	1
314.29	3	1
57.14	3	2
242.86	3	3
71.43	3	0
90	2	0
350	2	0
228.57	2	1
500	2	0
190	2	1
271.43	1	0
71.42	1	0
25	1	0
130	1	0
85.7	1	0
175	1	0
187.5	1	1
75	1	0
160	1	0

Table 5: **Complete set of experiments on the hydroxychloroquine set for zero- and few-shot in-context learning.**

		GPT-3.5			GPT-4		
Method	Shots (n)	MAE (mg/day)	SD (mg/day)	Within 0.1 mg/day (%)	MAE (mg/day)	SD (mg/day)	Within 0.1 mg/day (%)
Zero-shot	0	42.3	82.7	63.0	32.5	146.1	76.9
Word Count	1	37.3	89.6	64.8	49.2	169.6	73.6
Word Count	5	35.2	79.9	67.0	28.8	98.0	78.0
Word Count	10	26.7	52.7	71.0	12.3	49.0	86.4
Word Count	20	22.7	47.7	70.7	8.5	31.3	88.4
Word Count	40	23.3	48.2	74.3	6.4	25.1	89.9
Word Count	60	23.0	54.4	76.5	5.4	23.8	91.4
Word Count	75	22.6	54.1	77.5	5.6	23.5	91.4
Word Count	100	19.4	49.4	80.2	4.3	22.2	94.0
Uncommon Words	1	47.9	111.7	64.8	41.2	143.2	74.6
Uncommon Words	5	40.9	92.2	64.7	23.2	104.2	80.6
Uncommon Words	10	34.1	68.0	66.0	21.2	98.4	81.6
Uncommon Words	20	37.2	62.3	62.8	15.6	88.3	83.9
Uncommon Words	40	40.3	64.3	62.8	10.5	43.1	87.8
Uncommon Words	60	24.9	55.8	73.7	7.4	27.4	88.9
Uncommon Words	75	23.7	56.0	75.6	6.9	28.1	91.1
Uncommon Words	100	19.5	52.2	79.9	6.6	31.5	92.2
Model Disagreement	1	53.2	135.4	64.1	46.7	162.4	75.7
Model Disagreement	5	30.3	60.7	67.6	19.7	53.6	76.9
Model Disagreement	10	30.2	63.1	68.5	12.4	38.2	82.5
Model Disagreement	20	28.5	61.1	70.1	9.4	32.5	86.4
Model Disagreement	40	30.1	59.2	70.5	7.0	28.6	90.5
Model Disagreement	60	22.7	51.5	76.8	7.5	32.8	90.5
Model Disagreement	75	27.5	56.9	73.4	6.4	30.3	92.3
Model Disagreement	100	30.1	60.6	72.4	6.0	26.5	92.0
Random	1	40.8	84.5	62.6	23.4	58.6	76.9
Random	5	40.8	63.6	59.4	22.7	60.3	77.9
Random	10	45.4	71.3	57.9	18.6	48.7	81.2
Random	20	36.6	64.1	65.5	12.2	36.5	84.0
Random	40	32.6	57.7	64.2	11.1	35.3	84.9
Random	60	31.1	59.1	68.2	9.5	30.3	86.8
Random	75	19.1	46.1	78.0	7.6	29.6	89.8
Random	100	21.9	50.5	76.2	8.4	32.3	89.8
